# Circular RNA hsa_circ_0011324 is involved in endometrial cancer progression and the evolution of its mechanism

**DOI:** 10.1080/21655979.2022.2049026

**Published:** 2022-03-08

**Authors:** Dajiang Liu, Xuehan Bi, Yongxiu Yang

**Affiliations:** Department of Obstetrics and Gynecology, The first Hospital of Lanzhou University, Gansu, Lanzhou, China

**Keywords:** Hsa_circ_0011324, hsa-miR-497-5p, hsa-miR-16-5p, mechanistic target of rapamycin kinase, endometrial cancer

## Abstract

Endometrial cancer (EC) is one of the most common gynecological tumors with an increasing incidence. CircRNA plays an essential regulatory role in EC. Our objective was to investigate the potential mechanism of circRNAs derived SPOC Domain Containing 1 (SPOCD1) in EC progression. Seven circRNAs from SPOCD1 were analyzed by circBase and their expression was verified by quantitative real-time polymerase chain reaction. Only the expression of hsa_circ_0011324 was significantly increased in cancer tissues. The cell lines Ishikawa and RL95-2 which interfered with or overexpressed hsa_circ_0011324 were constructed and cell functions were tested. Results revealed hsa_circ_0011324 overexpression promoted cell proliferation, migration, and invasion; while silence of hsa_circ_0011324 had opposite effect on cell functions. RNA22 website and Targetscan website were applied to analyze downstream genes regulated by hsa_circ_0011324. Then, the expression of downstream genes was detected in EC tissues. Results indicated hsa-miR-497/16-5p expression were down-regulated, and mechanistic target of rapamycin kinase (mTOR) was up-regulated in EC. Furthermore, hsa_circ_0011324 regulated mTOR expression and cell functions by affecting hsa-miR-497/16-5p. And the potential mechanism was hsa_circ_0011324 competes with mTOR to directly bind to hsa-miR-497/16-5p. In conclusion, hsa_circ_0011324 could sponge hsa-miR-497/16-5p targeted mTOR to participate in EC progress. Our study may provide a new therapeutic target for EC.

## GRAPHICAL ABSTRACT

**Figure:**
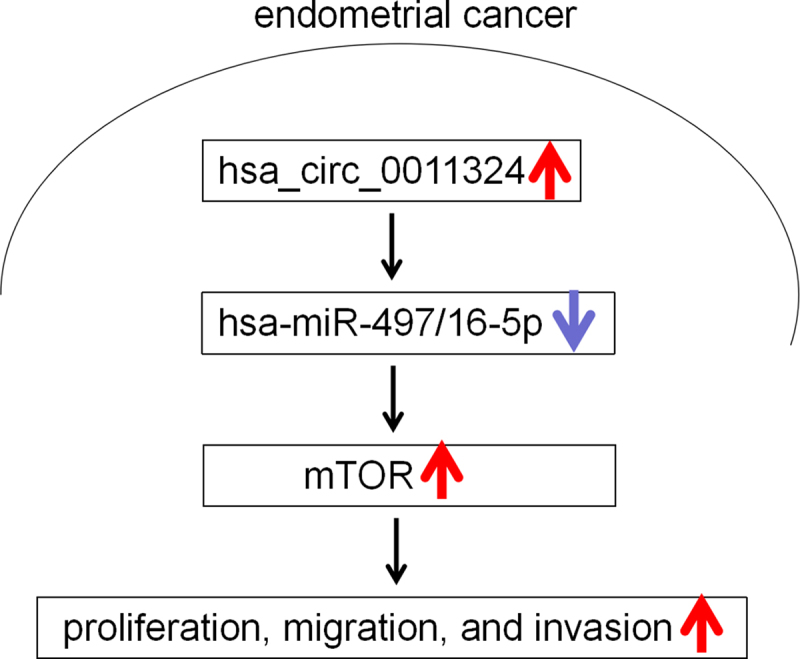

## Introduction

Endometrial cancer (EC) is one of the most common gynecological malignancies [1]. In recent years, under aging population, increasing obesity and diabetes influence, EC incidence has gradually increased [2], and the age of onset of EC has gradually become younger [3]. Endometrium changes under a variety of risk factors. Some non-physiological hyperplastic endometrium can be reverted to normal endometrium [4], while some are precancerous lesions and develop into EC of different pathological types [5]. The 5-year survival rate of EC patients decreased with EC stage development. The 5-year survival rate of 80% women diagnosed with EC stage I was >95% [6], while the 5-year survival rate of advanced patients was <20% [7]. Early detection and timely treatment are the key to improve patient’s prognosis. At present, there is no method to detect precancerous lesions or early cancers in time. Therefore, to explore a simple, accurate and economical screening method has become the focus of current research.

Circular RNA (circRNA) is a special class of non-coding RNA molecules (sometimes expressed in vivo), which is also the latest research hotspot in RNA field [8,9]. Different from traditional linear RNA (linear RNA with 5’ and 3’ ends), circRNA molecules have a closed ring structure and are not affected by RNA exonuclease, so their expression is more stable and not easy to degrade [10,11]. In terms of function, recent studies have shown that circRNA molecules are rich in microRNA (miRNA) binding sites and act as miRNA sponges in cells, thereby lifting miRNA inhibition on target genes and increasing target genes expression. This mechanism is known as competing endogenous RNAs (ceRNAs) mechanism [12]. J Jia Y, et al. reported circRNA hsa_circRNA_0001776 down-regulated leucine rich repeats and immunoglobulin like domains 2 and inhibited EC proliferation and promoted apoptosis through sponging miR-182 [13]. Wu B, et al. also reported a novel tumor regulator hsa_circ_0075960 acted as a sponge for miR-361-3p/SH2B adaptor protein 1 in EC cells and regulated EC progression by regulating miR-361-3p [14]. This indicated that circRNA played a vital regulatory role in EC through interactions with EC-associated miRNAs.

Our published papers have shown that SPOC Domain Containing 1 (SPOCD1) accelerated ovarian cancer progression and inhibited apoptosis through PI3K/AKT pathway [15]. We speculated the circRNAs that come from SPOCD1 also participate in EC progression. In this project, 7 circRNAs (hsa_circ_0011324, hsa_circ_0011325, hsa_circ_0011326, hsa_circ_0011327, hsa_circ_0011328, hsa_circ_0011329, hsa_circ_0011,330) from SPOCD1 were further analyzed by circBase. We used quantitative real-time polymerase chain reaction (qRT-PCR) to verify the differential expression of 7 circRNAs from SPOCD1, and found that hsa_circ_0011324 had significant differential expression. However, whether hsa_circ_0011324 participated in EC development and its specific mechanism has not been studied yet. Therefore, based on the above background, in this research, we investigated the mechanism of hsa_circ_0011324 in EC progression. Our goal is to provide a new strategies and targets for EC treatment.

## Materials and methods

### Collection of clinical samples

Sixty-four samples of cancer (CA) and para-cancerous (Para-CA) tissues came from patients with endometrial cancer in The First Hospital of Lanzhou University. None of the enrolled patients received chemotherapy or radiotherapy before surgery. The diagnosis and identification of EC were based on the standards of the World Health Organization. This study was approved by the institutional ethical board of The First Hospital of Lanzhou University (LDYYLL2021-99), and specimens were collected after each patient’s informed consent was obtained. The main clinical and pathological characteristics of these patients were collected. All patients were followed up for 5 years.

### Sanger sequencing and RT-qPCR

Sanger sequencing was performed according to previously reported methods [16,17]. In our study, RNA was extracted from samples of CA and Para-CA using TriQuick Reagent (R1100, Solarbio), which was reversed into complementary DNA (cDNA) using HiScript III RT SuperMix for qPCR (+gDNA WIper) (R323-01, Vazyme). The primers of hsa_circ_0011324 in Table 1 were used for amplification according to the protocol of 2× Taq Master Mix (P111-03, Vazyme). The amplified products were sequenced in Sangon biotech (Shanghai, China).

**Table 1.** The primers used in this study

Names	Sequences (5ʹto3’)
hsa_circ_0011324-F	GGAGAGGATGGTCAGCCCTA
hsa_circ_0011324-R	AGACCTGGGGAAAGGAGAGG
hsa_circ_0011325-F	GCCCTGTTTGAACTTCCTGTTT
hsa_circ_0011325-R	TTGTGGGTCATTTTGGAGGCT
hsa_circ_0011326-F	GCCCTGTTTGAACTTCCTGTT
hsa_circ_0011326-R	GGTGCCTTGTGTCAGGTCC
hsa_circ_0011327-F	CCTACCCACCTGCCCTGTTT
hsa_circ_0011327-R	TTTGGCTTAGGGCTTTCTGGA
hsa_circ_0011328-F	ATGGTCAGCCCTACCCACCT
hsa_circ_0011328-R	GGGTGACATCTCCATGAACCAC
hsa_circ_0011329-F	ATGGTCAGCCCTACCCACC
hsa_circ_0011329-R	CCCCAATATACAGGGATGGCTT
hsa_circ_0011330-F	TACCCACCTGCCCTGTTTGA
hsa_circ_0011330-R	AGGTAGCACACAAGCTTCACT
hsa-miR-497-5p-RT	GTCGTATCCAGTGCAGGGTCCGAGGTATTCGCACTGGATACGACACAAAC
hsa-miR-497-5p-F	CAGCAGCACACTGTG
hsa-miR-16-5p-RT	GTCGTATCCAGTGCAGGGTCCGAGGTATTCGCACTGGATACGACCGCCAA
hsa-miR-16-5p-F	TAGCAGCACGTAAATA
mTOR-F	AGCCGGAATGAGGAAACCAG
mTOR-R	TCATTGGAGGGGAGGAGGTT
U6-F	CTCGCTTCGGCAGCACA
U6-R	AACGCTTCACGAATTTGCGT
GAPDH-F	GAGTCAACGGATTTGGTCGT
GAPDH-R	GACAAGCTTCCCGTTCTCAG

In our study, we evaluated hsa_circ_0011324, hsa_circ_0011325, hsa_circ_0011326, hsa_circ_0011327, hsa_circ_0011328, hsa_circ_0011329, hsa_circ_0011330, hsa-miR-497-5p, hsa-miR-16-5p, and mTOR expression using qRT-PCR according to previously reported methods [16]. 2^−ΔΔCt^ was used to detect gene expression. U6 or GAPDH as internal reference. Table 1 showed the primers used in this study.

### Fluorescence in situ hybridization (FISH)

We performed FISH according to previous research [18]. In brief, precooled 4% paraformaldehyde (PFA) was placed and fixed at 4°C for 40 min. PFA was aspirated at room temperature and washed twice with a film breaker (0.1% Triton X-100 in phosphate-buffered saline (PBS)), 5 min each time. The probe was dropped onto the slipper, the slipper was sealed, desaturated at 85°C for 5 min, then taken out and hybridized overnight in an oven at 37°C and stained with DAPI. Mounting Medium sealed the film and wait until the Mounting Medium was solidified before taking pictures. The probe sequences used was as follows: hsa_circ_0011324: AAAGGCCCAATTTTACCAAAT-CY5, NC: ATCAGTCATCGACAATTAAAC-CY5. They were synthesized by Generay Biotech (Shanghai, China).

### Cell culture and treatment

The EC cells Ishikawa (#CC1103) and RL95-2 (#CC1106) were derived from CellCook. Ishikawa culture conditions were RPMI 1640 (#11875, Gibco) supplemented with 15% fetal bovine serum (FBS). RL95-2 was cultured with DMEM/F12 (#11330, Gibco) supplemented with 10% FBS containing 5 μg/mL insulin (#HY-P0035, MCE).

Ishikawa and RL95-2 cell lines with stable interference or overexpression of hsa_circ_0011324 were constructed and cultured, which were divided into Ishikawa-shNC, Ishikawa-shCIRC, Ishikawa-LVNC and Ishikawa-LVCIRC groups; RL95-2-shNC, RL95-2-shCIRC, RL95-2-LVNC, RL95-2-LVCIRC groups according to requirements. Interference and overexpression of hsa_circ_0011324 and its control virus were prepared by Hanbio Biotechnology (Shanghai, China).

To investigate whether hsa_circ_0011324 regulate EC progression through hsa-miR-497-5p and hsa-miR-16-5p, Ishikawa and RL95-2 cell lines with stable overexpression of hsa_circ_0011324 were transfected with hsa-miR-497-5p mimics and hsa-miR-16-5p mimics. The mimics of hsa-miR-497-5p or hsa-miR16-5p and their controls were provided by GenePharma (Shanghai, China). The above cell transfection steps were followed by Lipofectamine™ 2000 (BL623B, Biosharp) protocol.

### Cell counting Kit-8 (CCK-8) assay

Cell proliferation was determined using CCK-8 assay kit (G4103, Servicebio) as previous study [19]. In simple terms, cells in the logarithmic growth phase were taken, rinsed once with PBS, and digested by 0.25% trypsin into a single cell suspension. After digestion was terminated in complete medium, the cells were suspended in complete medium. Cells were counted, and the cell concentration was adjusted to 3 × 10^4^ cells/mL, and then inoculated into 96 well plates with 100 μL per well that was 3 × 10^3^ cells/well. Each cell line was tested for three multiple wells per day. 100 μL of complete medium was added to each well, and culture was continued at 37°C in a 5%CO_2_ incubator. After 12, 24, 48, 72 and 96 h, CCK-8 reagent was added in the ratio of 1:10 that was 100 μL culture solution was added into 10 μL detection solution. After incubation at 37°C for 2 h, the absorbance value at 450 nm was measured by a microplate reader.

### Clone formation assay

Clone formation assay was conducted as previous study method [20]. After cell counting, the cells were diluted to 1 × 10^4^cell/mL, and then diluted to 1 × 10^3^cell/mL and inoculated into 6-well plates. After inoculation, we added medium to 2 mL/well for each well, shook the culture plate from left to right in horizontal position to make the cells in the hole as evenly as possible. After one to two weeks of culture, the medium was removed from each well, rinsed with PBS or normal saline twice, fixed with 4% PFA for 15 min, rinsed with PBS twice, and 200 μL crystal violet staining solution was added to each well. After 20 min, the 6-well plate was rinsed under running water. After rinsing, the number of colonies was calculated.

### Transwell migration assay

As previous study method, we performed Transwell migration assay [20]. Cells in logarithmic growth phase were taken and rinsed once with PBS, then digested by 0.25% trypsin into single cell suspension. Appropriate amount of cell suspension was taken and centrifuged at 800 rpm for 5 min. After supernatant was aspirated and cells were suspended with basal medium, cell concentration was adjusted to 1 × 10^6^/mL with basal medium, 100 μL was added to upper chamber of Transwell chamber, and 600 μL of complete medium was added to lower chamber. Cells in upper chamber were wiped with cotton swabs, fixed with 4% PFA for 15 min, stained with 1% crystal violet for 10 min, and observed whether the cells passed through the pores under a microscope.

### Transwell invasion assay

As previous study method, we performed Transwell invasion assay [20]. Matrigel was dissolved overnight at 4°C, diluted with Matrigel: medium (1:3) in precooled basal medium, 40 μL was added to precooled Transwell chamber, the action should be slow to avoid bubbles. Matrigel was solidified after incubation at 37°C for 2 h. We added 100 μL and 600 μL basal medium to the upper and lower chambers, hydrated overnight at 37°C. The next day the medium was aspirated. After trypsin digestion, an appropriate amount of cell suspension was taken and centrifuged at 800 rpm for 5 min. After supernatant was aspirated and cells were suspended with basal medium, cell concentration was adjusted to 1 × 10^6^/mL with basal medium, 100 μL was added to upper chamber of Transwell chamber, and 600 μL of complete medium was added to lower chamber. The cells in upper chamber were wiped with cotton swabs, fixed with 4% paraformaldehyde for 15 minutes, stained with 1% crystal violet for 10 minutes, observed under a microscope and photographed.

### Immunohistochemistry (IHC)

We performed IHC following with previous study method [20]. First, xylene and ethanol were used for dewaxing and hydration. Then, fresh 3% H_2_O_2_ was prepared with distilled water or PBS and sealed at room temperature for 5–10 min. After that, it was heated by boiling and heated by electric furnace or water bath. Five percent BSA was used and sealed at room temperature for 15 minutes. We discarded the excess liquid and dropped mTOR Monoclonal Antibody (66,888-1-IG, Proteintech) overnight at 4°C. Polymerized HRP labeled anti-rabbit/mouse IgG secondary antibody was incubated. DAB was used for color rendering. Hematoxylin was redyed and washed twice with distilled water for 2 min each. Finally, after dehydration, transparent neutral resin was sealed and stored at room temperature.

### Western blot analysis

We detected protein expression via western blot analysis following with previous study [20]. Total protein was extracted with RIPA lysate. Protein was quantified according to BCA protein determination Kit (BL521A, Biosharp). SDS-PAGE loading buffer was mixed, and the mixture was heated in a boiling water bath at 100°C for 5 minutes. The protein was adsorbed on PVDF membrane by gel electrophoresis and sealed with 5% skim milk solution at room temperature for 90 min. mTOR Monoclonal Antibody (66,888-1-IG, Proteintech) and GAPDH (AB9485, ABCAM) were incubated overnight. The second antibody was incubated. Exposure was performed using an ultra-sensitive ECL chemiluminescence substrate (BL520A, Biosharp). GAPDH was acted as an internal reference to detect expression levels.

### RNA immunoprecipitation (RIP) assay

In our study, we used AGO2-RIP assay to confirm whether hsa_circ_0011324 can compete with mTOR to bind to miRNA [21]. Ishikawa and RL95-2 cell lines with stable overexpression of hsa_circ_0011324 were cultured and divided into Ishikawa-LVNC, Ishikawa-LVCIRC, RL95-2-LVNC and RL95-2-LVCIRC groups according to requirements. The cells were collected for RIP test. After RNA was obtained, hsa_circ_0011324 and mTOR mRNA expressions were evaluated by qRT-PCR. Using the Magna RIP™ RNA-Binding Protein Immunoprecipitation Kit (#17-700) provided by Millipore, RIP was performed according to the instructions. The antibody used was Anti-Argonaute-2 antibody [EPR10411] (ab186733, ABCAM).

### Bioinformatics prediction and dual-luciferase assay

RNA22 website and Targetscan website were used to analyze downstream genes regulated by hsa_circ_0011324. Then, dual-luciferase assay was conducted following with the method in previous study [20]. 293 T cells were purchased from CellCook (#CC4003) and cultured in DMEM high Glucose (#11995, Gibco) with 10% FBS. According to the TransDetect® Double-Luciferase Reporter Assay Kit (FR201-01, TransGen Biotech), the binding effect of hsa_circ_0011324 with hsa-miR-497/16-5p and the binding effect of hsa-miR-497/16-5p with mTOR mRNA 3ʹUTR (untranslated regions) were detected. Fluorescence reporter plasmid was synthesized by Genecfps (Jiangsu, China).

### Statistical analysis

Graphpad Prism 8.0 software was used for statistical analysis. Measurement data were expressed as mean ± standard deviation (SD). Student’s t-test was used between the two groups, and one-way analysis of variance (ANOVA) was performed for inter-group comparison. The disease-free survival (DFS) and overall survival (OS) rates were calculated through Kaplan-Meier, and differences were compared using a log-rank test. chi-square (and Fisher’s exact) test detected the relationship between hsa_circ_0011324 and clinicopathological parameters. Cox regression (Univariate and multivariate) analyzed OS in EC patients. Pearson Correlation Coefficient studied the correlation between hsa_circ_0011324 and hsa-miR-497-5p, hsa_circ_0011324 and hsa-miR-16-5p. P < 0.05 indicated the difference was statistically significant.

## Results

Our published papers have shown that SPOCD1 accelerated ovarian cancer progression. We speculated the circRNAs that come from SPOCD1 also participate in EC progression. In this project, 7 circRNAs (hsa_circ_0011324, hsa_circ_0011325, hsa_circ_0011326, hsa_circ_0011327, hsa_circ_0011328, hsa_circ_0011329, hsa_circ_0011330) from SPOCD1 were found in circBase. We found that only hsa_circ_0011324 had significant differential expression in EC via qRT-PCR detection. Therefore, based on the above background, in this research, we investigated the mechanism of hsa_circ_0011324 in EC progression. Results showed that hsa_circ_0011324 could sponge hsa-miR-497/16-5p targeted mTOR to participate in EC progress.

## 1. Hsa_circ_0011324 expression in EC and its correlation with prognosis

**Figure 1.**
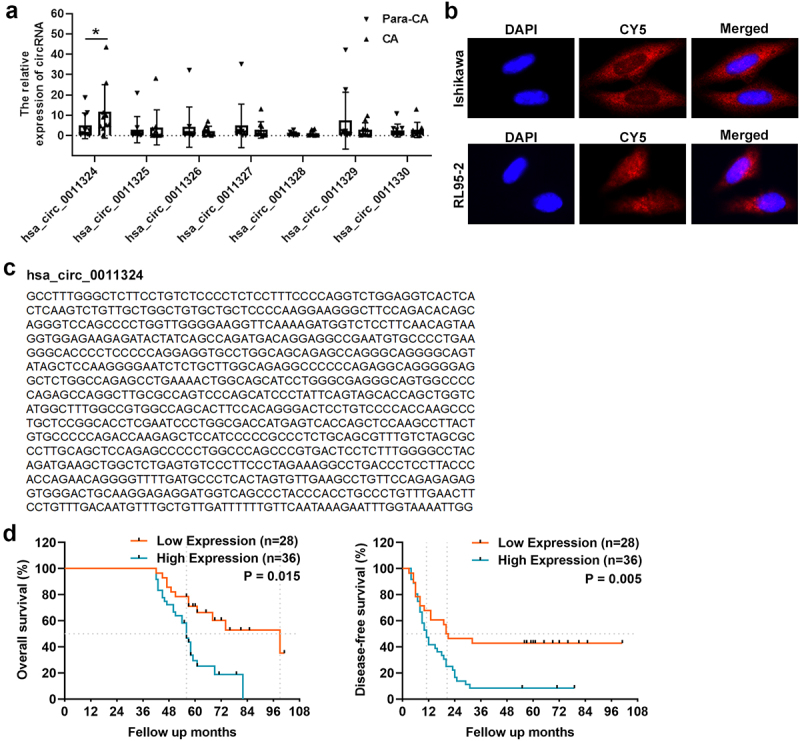
Hsa_circ_0011324 expression in endometrial cancer and its correlation with prognosis. (a). Quantitative real-time polymerase chain reaction analysis of SPOCD1-derived circRNAs (hsa_circ_0011324, hsa_circ_0011325, hsa_circ_0011326, hsa_circ_0011327, hsa_circ_0011328, hsa_circ_0011329, and hsa_circ_0011330) expression in CA (cancer tissue) and Para-CA (para-cancerous) came from patients with endometrial cancer. (b). Left is the sequence of hsa_circ_0011324 from circBase, right is the Sanger sequencing analysis to confirm the loop structure of hsa_circ_0011324. (c). Fluorescence in situ hybridization experiment detected the localization of hsa_circ_0011324 in endometrial cancer cell lines (Ishikawa and RL95-2). (d). Kaplan-Meier survival curves for endometrial cancer patients according to hsa_circ_0011324 expression level. * P < 0.05.

First, we used CA and Para-CA samples to examine SPOCD1-derived circRNAs (hsa_circ_0011324, hsa_circ_0011325, hsa_circ_0011326, hsa_circ_0011327, hsa_circ_0011328, hsa_circ_0011329, and hsa_circ_0011330) expression using qRT-PCR. Only hsa_circ_0011324, which originated from exon 16 of the SPOCD1 gene, was significantly increased in CA (Figure 1a). Therefore, we selected hsa_circ_0011324 for further study. Firstly, we searched circBase to find the sequence of hsa_circ_0011324 (Figure 1b left). Then Sanger sequencing was used to confirm the loop structure of hsa_circ_0011324, and results indicated the head-to-tail splice site was consistent with the sequence from circBase (Figure 1b right). Next, FISH experiment was used to evaluate cellular localization of hsa_circ_0011324 in EC cell lines (Ishikawa and RL95-2). And results demonstrated hsa_circ_0011324 was mainly localized in cytoplasm (Figure 1c), indicated that hsa_circ_0011324 may participate in EC progression through ceRNAs mechanism. Afterward, we verified the hsa_circ_0011324 expression and its correlation with prognosis in EC tissues. Kaplan–Meier survival curves showed the high expression of hsa_circ_0011324 in EC tissues was closely related to poor OS and DFS (Figure 1d). Table 2 showed the relationship between hsa_circ_0011324 and clinicopathological parameters. Among them, hsa_circ_0011324 was closely related to tumor staging. Table 3 showed the univariate and multivariate analysis of OS in EC patients. Among them, circRNA expression and Clinical Stage FIGO had significant differences.

**Table 2.** Relationship between hsa_circ_0011324 expression and clinicopathological parameters

Variable	Total	High expression	Low expression	*P* value
Age (year)				0.748
≤45	17	9	8	
>45	47	27	20	
Pathologic differentiation				0.010*
Adenocarcinoma	45	30	15	
Non-adenocarcinoma	19	6	13	
BMI				0.032*
≥28	21	16	5	
<28	43	20	23	
Pregnancy				0.963
Yes	55	31	24	
No	9	5	4	
Menopause				0.073
Yes	31	21	10	
No	33	15	18	
Grade				0.0483*
1	24	10	15	
2	28	16	10	
3	12	10	3	
LN metastasis				0.042*
negative	46	21	23	
positive	18	15	5	
Clinical Stage (FIGO)				0.014*
I	21	8	13	
II	19	9	10	
III–IV	24	19	5	

*: Chi-square (and Fisher’s exact) test, P value<0.05.

A: American Joint Committee on Cancer (AJCC), patients were staged in accordance with the 8th Edition of the AJCC Cancer’s TNM Classification.

**Table 3.** Univariate and multivariate analysis of Overall Survival (OS) in EC patients (n = 64)

Variables	Univariate analysis			Multivariate analysis		
	HR	95% CI	P-Value	HR	95% CI	P-Value
Age (<45 vs>45)	1.07	1.03–1.11	0.001**	1.02	0.94–1.12	0.585
Pathological (Adenocarcinoma vs non-adenocarcinoma)	1.17	1.08–1.27	0.000***	1.05	0.95–1.24	0.054
BMI (<28 vs >28)	1.17	1.08–1.27	0.000***	1.1	0.99–1.23	0.067
Pregnancy (Yes vs No)	0.81	0.34–1.95	0.641			
Menopause (Yes vs No)	2.13	1.10–4.11	0.025*	0.68	0.19–2.37	0.544
CircRNA expression (High vs Low)	2.9	1.41–5.96	0.004**	2.46	1.12–5.39	0.025*
Grade	1.77	1.16–2.27	0.009**	0.6	0.27–1.32	0.026
Lymphatic metastasis (Yes vs No)	3.89	1.93–7.85	0.000***	1.36	0.52–3.59	0.53
Clinical Stage FIGO (I vs. II vs III, Ⅳ)	2.09	1.48–2.95	0.000***	2.1	1.06–4.19	0.034*

Abbreviations: HR hazard ratio, 95%CI 95% confidence interval, Cox regression analysis, * p < 0.05, ** p < 0.01, *** p < 0.001.

## 2. Hsa_circ_0011324 promoted EC progression

**Figure 2.**
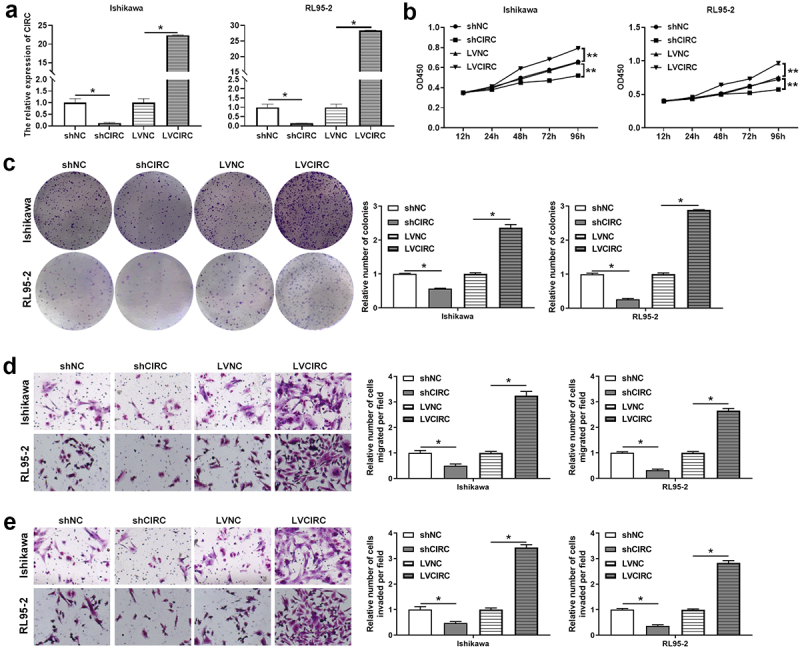
Hsa_circ_0011324 promoted endometrial cancer progression. (a). Quantitative real-time polymerase chain reaction determined hsa_circ_0011324 expression changes in Ishikawa and RL95-2 cells after stable overexpression or interference with hsa_circ_0011324. After stable overexpression or interference with hsa_circ_0011324 in Ishikawa and RL95-2 cells, (b) Cell Counting Kit-8 evaluated cell proliferation changes. (c). Clone formation detected the number of cell clones. (d) and (e). Transwell measured cell migration (d) and invasion (e) changes. CIRC indicates hsa_circ_0011324; shNC indicates the negative control of interference with hsa_circ_0011324; shCIRC indicates interference with hsa_circ_0011324; LVNC indicates the negative control of hsa_circ_0011324 overexpression; LVCIRC indicates hsa_circ_0011324 overexpression. * P < 0.05, ** P < 0.01.

Figure 1 indicated that hsa_circ_0011324 maybe participate in the EC progression. Therefore, we further studied the hsa_circ_0011324 function in EC cell lines (Ishikawa and RL95-2). Firstly, Ishikawa and RL95-2 cell lines that interfere with or overexpress hsa_circ_0011324 were constructed. Figure 2a showed the cell lines Ishikawa and RL95-2 that interfered with or overexpressed hsa_circ_0011324 were successfully constructed. After that, we performed cell function tests on Ishikawa and RL95-2 cells. Hsa_circ_0011324 overexpression promoted cell proliferation, migration, and invasion (Figure 2b-2E). Silence of hsa_circ_0011324 inhibited cell proliferation, migration, and invasion (Figure 2b-2E). Base on the above results, we found that hsa_circ_0011324 overexpression promoted EC progression, while knockdown of hsa_circ_0011324 inhibited EC progression.

## 3. Hsa-miR-497/16-5p were down-regulated while mTOR was up-regulated in EC tissues

**Figure 3.**
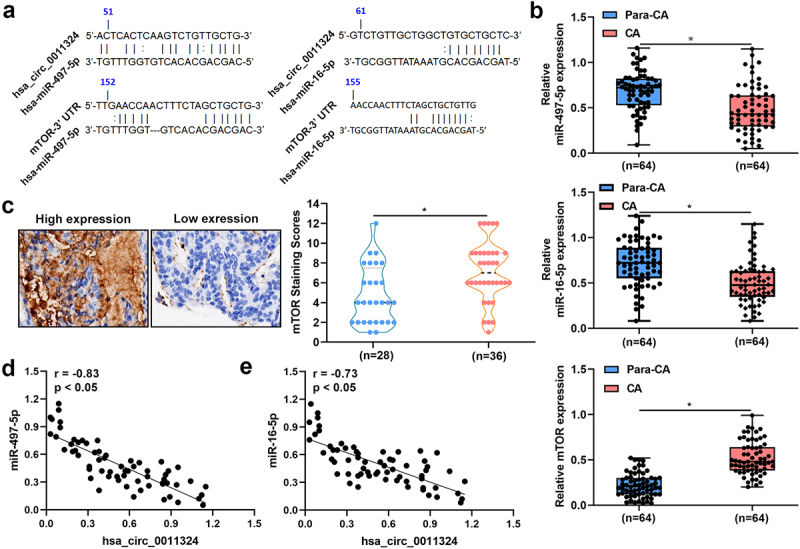
Hsa-miR-497/16-5p were down-regulated while mTOR was up-regulated in endometrial cancer tissues. (a). RNA22 website combined with targetscan website were used to analyze the potential downstream genes of hsa_circ_0011324. (b). Quantitative real-time polymerase chain reaction detected hsa-miR-497-5p, hsa-miR-16-5p, and mTOR mRNA expressions in CA (cancer tissue) and Para-CA (para-cancerous) came from patients with endometrial cancer. (c). Immunohistochemistry evaluated mTOR expression. (d). Pearson Correlation Coefficient studied the correlation between hsa_circ_0011324 and hsa-miR-497-5p. (e). Pearson correlation coefficient studied the correlation between hsa_circ_0011324 and hsa-miR-16-5p. mTOR, mechanistic target of rapamycin kinase. * P < 0.05.

To study whether hsa_circ_0011324 regulated downstream genes to participate in EC progression, RNA22 website and Targetscan website were used to analyze the potential downstream genes of hsa_circ_0011324. Results indicated that hsa_circ_0011324 might inhibit the targeted inhibitory effect of hsa-miR-497-5p and hsa-miR-16-5p on mTOR by sponging hsa-miR-497-5p and hsa-miR-16-5p (Figure 3a). Figure 3b and 3C showed the expression of hsa-miR-497-5p and hsa-miR-16-5p was low, while mTOR expression was high in CA compared to Para-CA. Pearson Correlation Coefficient revealed hsa-miR-497-5p and hsa-miR-16-5p expressions in EC was negatively correlated with hsa_circ_0011324 (Figure 3d and 3E). These results suggested that the downstream genes (hsa-miR-497/16-5p and mTOR) involved in EC progression.

## 4. Hsa_circ_0011324 regulated mTOR expression by affecting hsa-miR-497/16-5p

**Figure 4.**
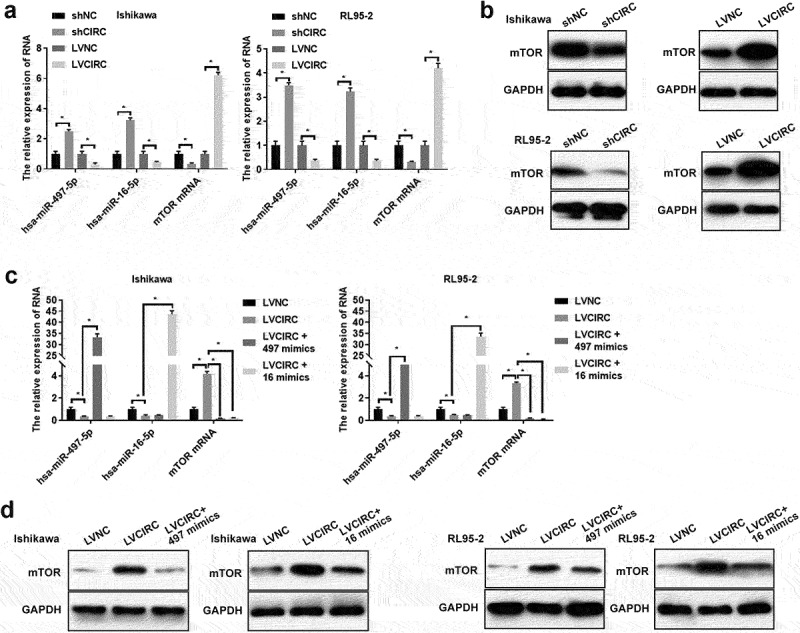
Hsa_circ_0011324 regulated mTOR expression by affecting hsa-miR-497/16-5p. After stably overexpression or interference with hsa_circ_0011324 in Ishikawa and RL95-2 cells, (a). Quantitative real-time polymerase chain reaction determined hsa-miR-497/16-5p and mTOR mRNA expression. (b). Western blotting detected mTOR protein expression. After overexpression of hsa_circ_0011324 and then overexpression of hsa-miR-497/16-5p in Ishikawa and RL95-2 cells, (c). Quantitative real-time polymerase chain reaction evaluated hsa_circ_0011324, hsa-miR-497/16-5p, and mTOR mRNA expression. (d) Western blotting assessed mTOR protein expression. mTOR indicates mechanistic target of rapamycin kinase; shNC indicates the negative control of interference with hsa_circ_0011324; shCIRC indicates interference with hsa_circ_0011324; LVNC indicates the negative control of hsa_circ_0011324 overexpression; LVCIRC indicates hsa_circ_0011324 overexpression; LVCIRC+497 mimics indicates overexpression of hsa_circ_0011324 and hsa-miR-497-5p; LVCIRC+16 mimics indicates overexpression of hsa_circ_0011324 and hsa-miR-16-5p. * P < 0.05.

Previous researches confirmed hsa-miR-497-5p mimics and hsa-miR-16-5p mimics decreased mTOR expression [22–24]. Therefore, we further studied whether hsa_circ_0011324 regulated mTOR expression via hsa-miR-497/16-5p in EC progression. We firstly detected hsa-miR-497/16-5p and mTOR expression in cell lines Ishikawa and RL95-2 that stably overexpress or interfere with hsa_circ_0011324. Figure 4a and 4B showed overexpression of hsa_circ_0011324 inhibited hsa-miR-497/16-5p expression and promoted mTOR expression; while interference with hsa_circ_0011324 promoted hsa-miR-497/16-5p expression and inhibited mTOR expression. Next, we overexpressed hsa-miR-497/16-5p in cell lines Ishikawa and RL95-2 that stably overexpress hsa_circ_0011324. Figure 4c showed that the overexpression of hsa-miR-497/16-5p was successful. In addition, overexpression of hsa_circ_0011324 and then overexpression of hsa-miR-497/16-5p could inhibit the increase of mTOR expression caused by overexpression of hsa_circ_0011324 (Figure 4c and 4D). These results demonstrated that hsa_circ_0011324 regulated mTOR expression by affecting hsa-miR-497/16-5p.

## 5. Hsa_circ_0011324 was involved in EC progression by affecting hsa-miR-497/16-5p

**Figure 5.**
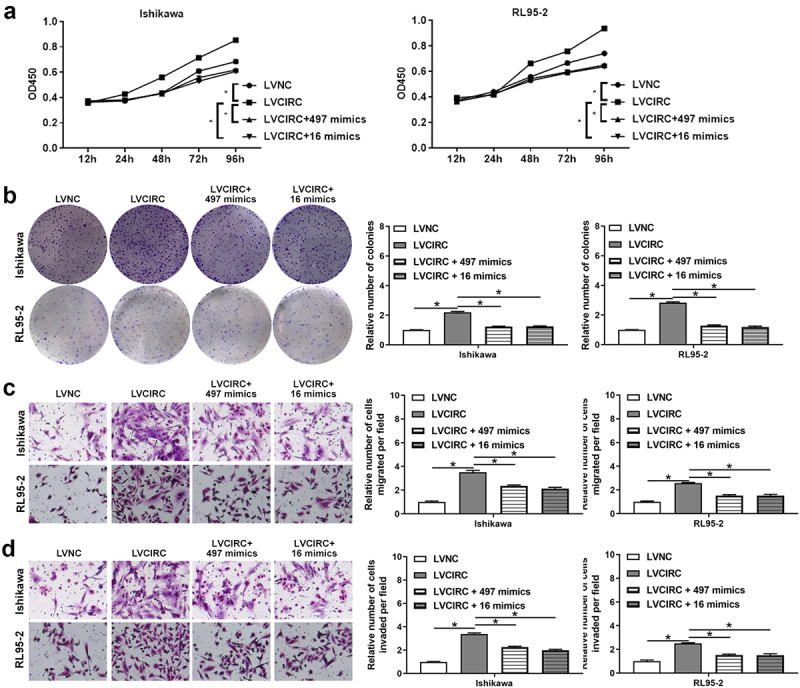
**Hsa_circ_0011324 was involved in endometrial cancer progression by affecting hsa-miR-497/16-5p**. After overexpression of hsa_circ_0011324 and then overexpression of hsa-miR-497/16-5p in Ishikawa and RL95-2 cells, (a). Cell Counting Kit-8 measured cell proliferation changes. (b). Clone formation assessed the number of cell clones. C and D. Transwell detected cell migration (c) and invasion (d) changes. LVNC indicates the negative control of hsa_circ_0011324 overexpression; LVCIRC indicates hsa_circ_0011324 overexpression; LVCIRC+497 mimics indicates overexpression of hsa_circ_0011324 and hsa-miR-497-5p; LVCIRC+16 mimics indicates overexpression of hsa_circ_0011324 and hsa-miR-16-5p. * P < 0.05.

Previous studies showed hsa-miR-497-5p and hsa-miR-16-5p expression were down-regulated in EC [25–27] . Therefore, we further investigated whether hsa_circ_0011324 was involved in EC progression by affecting hsa-miR-497/16-5p. After overexpression of hsa-miR-497/16-5p in Ishikawa and RL95-2 that stably overexpress hsa_circ_0011324, we performed cell function tests. Figures 5A and 5B showed hsa_circ_0011324 overexpression and then overexpression of hsa-miR-497/16-5p could inhibit the increase in cell proliferation and the number of cell clones caused by overexpression of hsa_circ_0011324. Besides, overexpression of hsa_circ_0011324 and then overexpression of hsa-miR-497/16-5p could inhibit hsa_circ_0011324 overexpression inducing enhance of cell migration and invasion (Figure 5c and 5D). These results showed hsa_circ_0011324 participated in EC progression by affecting hsa-miR-497/16-5p.

## 6. Hsa_circ_0011324 competes with mTOR to directly bind to hsa-miR-497/16-5p

**Figure 6.**
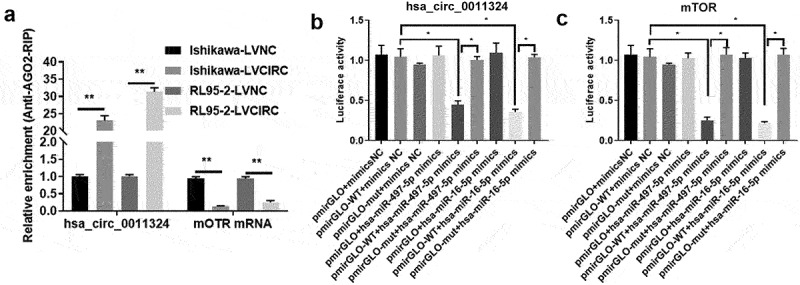
Hsa_circ_0011324 competes with mTOR to directly bind to hsa-miR-497/16-5p. (a). Overexpression of hsa_circ_0011324 in Ishikawa and RL95-2 cells, then conducted AGO2-RIP (RNA immunoprecipitation) followed with quantitative real-time polymerase chain reaction detection of hsa_circ_0011324 and mTOR. (b) Dual-luciferase assessed the binding effect of hsa_circ_0011324 and hsa-miR-497/16-5p. (c). Dual-luciferase tested the binding effect of hsa-miR-497/16-5p and mTOR mRNA 3ʹUTR (untranslated regions). LVNC indicates the negative control of hsa_circ_0011324 overexpression; LVCIRC indicates hsa_circ_0011324 overexpression; WT indicates wild type; mut indicates mutant type; mimics NC indicates the negative control of mimics. * P < 0.05, ** P < 0.01.

To confirm the relationship between hsa_circ_0011324, hsa-miR-497/16-5p, and mTOR, we performed AGO-RIP and Dual-luciferase assay. AGO2 can enrich hsa_circ_0011324 and mTOR, and overexpression of hsa_circ_0011324 decreased enrichment of mTOR on AGO2 (Figure 6a). Dual-luciferase results further proved hsa_circ_0011324 could directly sponge hsa-miR-497/16-5p (Figure 6b). At the same time, hsa-miR-497/16-5p could directly target mTOR mRNA (Figure 6c). These results illuminated that hsa_circ_0011324 competes with mTOR to directly bind to hsa-miR-497/16-5p.

## Discussion

EC often occurs in postmenopausal women, and endometrium is a well-known site of cancer affecting women [28]. Currently, EC treatment remains a challenge for gynecological and radiation oncologists [29]. CircRNA plays an essential regulatory role in EC. This project used circBase to analyze the differential expression of 7 circRNAs from SPOCD1, and found hsa_circ_0011324 had significant differential expression. Further, we investigated the mechanism of hsa_circ_0011324 and its regulated downstream genes in EC. Our study illuminated hsa_circ_0011324 could sponge hsa-miR-497/16-5p targeted mTOR to participate in EC progress. This is the first report on the progress and mechanism of hsa_circ_0011324ʹs participation in EC.

With the enhancement of RNA sequencing methods, more and more circRNAs have been identified and their functions have been gradually revealed [30]. In addition, several clinical trials are investigating the biomarkers and therapeutic effects of circRNAs. These efforts might provide a new perspective for gynecological tumors diagnosis and treatment. In addition, ceRNAs mechanism suggested circRNAs can regulate mRNA by competing with same pool miRNAs [31]. In recent years, researchers have reported circRNA-ceRNA mechanism in EC. For example, Wang Y, et al. found circRNA hsa_circ_002577 acted as a miR-625-5p sponge to up-regulate insulin like growth factor 1 receptor and activate PI3K/Akt pathway, thereby accelerating EC progression [32]. Zong ZH, et al. reported circ_PUM1 could compete with miR-136, leading to Notch receptor 3 upregulation, thereby promoting EC development [33]. Liu Y, et al. also found circ_0067835 could compete with miR-324-5p, leading to high mobility group AT-Hook 1 upregulation and thus inducing EC [34]. However, the molecular mechanism of hsa_circ_0011324 involved in EC is still unclear. In this study, we found that hsa_circ_0011324 was high expression in EC tissues, and the high expression of hsa_circ_0011324 was closely associated with poor OS/DFS. In addition, hsa_circ_0011324 overexpression promoted EC cell proliferation, migration, and invasion; while knockdown of hsa_circ_0011324 inhibited EC cell proliferation, migration, and invasion. These results indicated that hsa_circ_0011324 involved in EC progression.

Previous studies have shown that changes in hsa-miR-497-5p expression affected EC occurrence, and the low expression of miR-497-5p was related to high-risk EC [25,35]. In addition, hsa-miR-16-5p was down-regulated in EC and inhibited EC [27]. These findings suggested that hsa-miR-497-5p and hsa-miR-16-5p might be used as good predictive and distinguishing factors for EC, and may be used as potential biomarkers. In addition, miR-497-5p can as a sponge of circSLC6A6 to regulate tumor-related signaling pathway PI4KB/hedgehog and participate EC progress [36]. And long non-coding RNA SNHG25 sequestered miR-497-5p acted as ceRNA in EC, thereby positively regulating fatty acid synthase expression and promoting EC deterioration [26]. In this study, we found hsa-miR-497-5p and hsa-miR-16-5p were low expression in EC, and their expression in EC tissues were negatively correlated with hsa_circ_0011324. These results were consistent with the previously reportion that low expression trend of hsa-miR-497-5p and hsa-miR-16-5p in EC tissues. Moreover, our study suggested hsa_circ_0011324 overexpression promoted cell functions (proliferation, migration, and invasion) via affected the expression of hsa-miR-497/16-5p.

PI3K/AKT/mTOR pathway is a classical pathway in cancer, and this pathway inhibitors are effective and safe in patients with locally advanced, metastatic or recurrent EC [37,38]. Such as Barra F, et al. reported PI3K/AKT/mTOR signaling inhibitors had great clinical value in EC [39]. In this research, we found mTOR was highly expressed in EC. And hsa_circ_0011324 overexpression promoted mTOR expression by affected the expression of hsa-miR-497/16-5p. Furthermore, AGO2-RIP and dual-luciferase verified hsa_circ_0011324 competes with mTOR to directly bind to hsa-miR-497/16-5p. This is the first time we reported hsa_circ_0011324-hsa-miR-497/16-5p-mTOR axis participate in EC

However, the above mechanism was confirmed only in vivo cell experiments. More studies are needed in vitro to verify our conclusion. In the future studies, we will conduct tumorigenesis in nude mice to confirm whether hsa_circ_0011324 involve in EC progression by sponging with hsa-miR-497/16-5p to regulate mTOR; and more EC tissues will be collected to confirm whether hsa_circ_0011324 can be a predictor for EC diagnosis and treatment.

## Conclusion

Hsa_circ_0011324 was high expression in EC, which indicated poor OS and DFS. Overexpression of hsa_circ_0011324 promoted the ability of proliferation, migration, and invasion in EC cells; while silence of hsa_circ_0011324 had opposite effect on cell functions. Furthermore, we reported for the first time that hsa_circ_0011324 could sponge hsa-miR-497/16-5p targeted mTOR to participate in EC progress.

## Supplementary Material

Supplemental Material

## Highlights

- Hsa_circ_0011324 promoted EC progression.
- Hsa-miR-497/16-5p were low expression, while mTOR was high expression in EC.
- Hsa_circ_0011324 was involved in EC progression by affecting hsa-miR-497/16-5p.
- 4. Hsa_circ_0011324 competes with mTOR to directly bind to hsa-miR-497/16-5p.
