# Supplementary figures and images for: Circular RNA hsa_circ_0011324 is involved in endometrial cancer progression and the evolution of its mechanism
Source: Bioengineered. 2022 Mar 8;13(3):7485–99. doi: 10.1080/21655979.2022.2049026 (PMC8973664; doi:10.1080/21655979.2022.2049026)

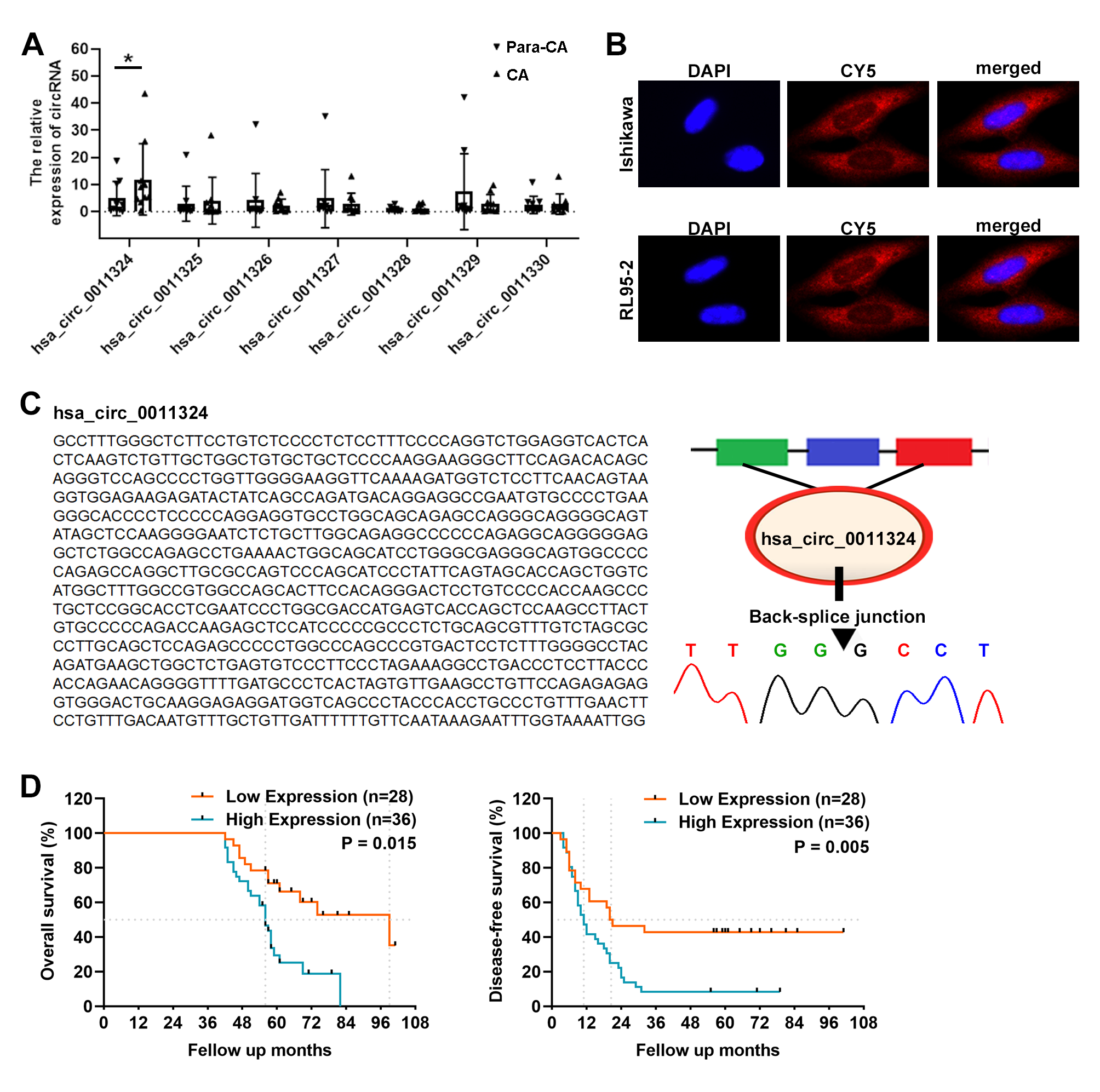

Supplement: Supplemental Material [file KBIE_A_2049026_SM0068.tif]
